# Stability of the Parainfluenza Virus 5 Genome Revealed by Deep Sequencing of Strains Isolated from Different Hosts and following Passage in Cell Culture

**DOI:** 10.1128/JVI.03351-13

**Published:** 2014-04

**Authors:** Bert K. Rima, Derek Gatherer, Daniel F. Young, Hanna Norsted, Richard E. Randall, Andrew J. Davison

**Affiliations:** a Centre for Infection and Immunity, School of Medicine, Dentistry and Biomedical Sciences, The Queen's University of Belfast, Belfast, United Kingdom; b MRC-University of Glasgow Centre for Virus Research, Glasgow, United Kingdom; c Centre for Biomolecular Sciences, School of Biology, University of St. Andrews, St. Andrews, United Kingdom

## Abstract

The strain diversity of a rubulavirus, parainfluenza virus 5 (PIV5), was investigated by comparing 11 newly determined and 6 previously published genome sequences. These sequences represent 15 PIV5 strains, of which 6 were isolated from humans, 1 was from monkeys, 2 were from pigs, and 6 were from dogs. Strain diversity is remarkably low, regardless of host, year of isolation, or geographical origin; a total of 7.8% of nucleotides are variable, and the average pairwise difference between strains is 2.1%. Variation is distributed unevenly across the PIV5 genome, but no convincing evidence of selection for antibody-mediated evasion in hemagglutinin-neuraminidase was found. The finding that some canine and porcine, but not primate, strains are mutated in the SH gene, and do not produce SH, raised the possibility that dogs (or pigs) may not be the natural host of PIV5. The genetic stability of PIV5 was also demonstrated during serial passage of one strain (W3) in Vero cells at a high multiplicity of infection, under conditions of competition with large proportions of defective interfering genomes. A similar observation was made for a strain W3 mutant (PIV5VΔC) lacking V gene function, in which the dominant changes were related to pseudoreversion in this gene. The mutations detected in PIV5VΔC during pseudoreversion, and also those characterizing the SH gene in canine and porcine strains, predominantly involved U-to-C transitions. This suggests an important role for biased hypermutation via an adenosine deaminase, RNA-specific (ADAR)-like activity.

**IMPORTANCE** Here we report the sequence variation of 16 different isolates of parainfluenza virus 5 (PIV5) that were isolated from a number of species, including humans, monkeys, dogs, and pigs, over 4 decades. Surprisingly, strain diversity was remarkably low, regardless of host, year of isolation, or geographical origin. Variation was distributed unevenly across the PIV5 genome, but no convincing evidence of immune or host selection was found. This overall genome stability of PIV5 was also observed when the virus was grown in the laboratory, and the genome stayed remarkably constant even during the selection of virus mutants. Some of the canine isolates had lost their ability to encode one of the viral proteins, termed SH, suggesting that although PIV5 commonly infects dogs, dogs may not be the natural host for PIV5.

## INTRODUCTION

A virus that was first isolated almost 6 decades ago from rhesus and crab-eating (cynomolgus) macaque kidney cells was originally named simian virus 5 (SV5) because it was believed that monkeys were its natural host (1, 2). However, wild monkeys do not have antibodies against SV5 and appear to be infected in captivity after contact with humans (3–5), who can be infected naturally (4, 6). Subsequently, it was shown that SV5 also causes kennel cough in dogs, and as a consequence, it is often referred to in veterinary circles as canine parainfluenza virus. In addition, the virus has been isolated from pigs, and there is some evidence that cats, hamsters, and guinea pigs can be infected (4, 7, 8). Because SV5 has been isolated from numerous species, and its natural host remains unidentified, it is now named parainfluenza virus 5 (PIV5).

Two issues have complicated studies on defining the host range and prevalence of PIV5. First, the virus can appear as a contaminant of tissue culture cells, raising the possibility that some strains may have been isolated accidentally. However, limited studies of sequence diversity among strains suggest that this is unlikely to have occurred frequently, if at all (7). Second, antigenic cross-reactivity occurs between PIV5 and human parainfluenza virus 2 (PIV2) (9, 10). This led to an early suggestion that PIV5 (or SV5, as it was called at the time) should be classified as PIV2 of monkeys (11). However, sequencing studies confirmed that PIV5 and PIV2 belong to distinct species (Parainfluenza virus 5 and Human parainfluenza virus 2, respectively; genus Rubulavirus, subfamily Paramyxovirinae, family Paramyxoviridae, order Mononegavirales) (12). Thus, for example, PIV5 hemagglutinin-neuraminidase (HN) shares only 43% amino acid sequence identity with its PIV2 ortholog (13).

PIV5 has a nonsegmented, negative-sense RNA genome of 15,246 nucleotides (nt). The genome contains seven genes, which encode eight proteins and are flanked by 3′-leader and 5′-trailer sequences at the genome ends. From the 3′ end, the genome encodes the nucleocapsid protein (N), V protein (V), phosphoprotein (P), matrix protein (M), fusion protein (F), small hydrophobic protein (SH), HN, and large protein (L) or RNA polymerase (8, 12). V and P are unusual in being encoded by a single gene (V/P) and sharing the same initiation codon and 5′-coding region. However, they differ in their 3′-coding regions, with the V mRNA being a faithful copy of the genomic sequence and the P mRNA being a frameshifted version generated during transcription by the pseudotemplated addition of 2 extra G residues in a G tract. The outcome is that the V and P proteins share an N-terminal domain of 164 amino acid residues but differ in their C-terminal domains (58 and 228 residues, respectively).

Complete PIV5 genome sequences have been deposited in public databases for the simian strain W3 (also called W3A), the human strain cryptovirus, and, recently, three canine strains and one porcine strain (Table 1). We have assessed PIV5 diversity by resequencing strain W3 and also determining the sequences of five human strains, three canine strains, and a porcine strain. In addition, we have investigated the stability of the PIV5 genome during passage in cell culture at a high multiplicity of infection (MOI) of strain W3 and a W3-derived mutant lacking a functional V gene. We found that the PIV5 genome exhibits low diversity both *in vivo* and *in vitro* and also that SH is not essential for infection of dogs or pigs.

**TABLE 1:** Information on the PIV5 genome sequences determined in this study (with read data) or deposited by others

PIV5 strain	Host source	Country of origin	Decade of isolation	GenBank accession no.	No. of Illumina reads	% matching reads
MEL	Human	UK	1970s	JQ743325	2,711,800	92
MIL	Human	UK	1970s	JQ743326	2,452,893	88
DEN	Human	UK	1970s	JQ743322	2,222,620	89
LN	Human	UK	1970s	JQ743324	2,229,468	83
RQ	Human	UK	1970s	JQ743327	2,070,127	77
W3(cl)	Macaque	USA	1960s	AF052755		
W3	Macaque	USA	1960s	JQ743318	21,156,178	78
Cryptovirus	Human	USA	Not known	AX586923		
SER	Pig	Germany	1990s	JQ743328	1,634,295	71
KNU-11	Pig	South Korea	2010s	KC852177		
CPI^+^	Dog	Germany	1980s	JQ743321	1,505,580	59
CPI^−^	Dog	Germany	1980s	JQ743320	1,441,355	64
1168-1	Dog	South Korea	2000s	KC237064		
78524	Dog	UK	1980s	JQ743319	2,294,166	78
H221	Dog	UK	1980s	JQ743323	2,251,985	86
08-1990	Dog	South Korea	2000s	KC237063		
D277	Dog	South Korea	2000s	KC237065		

## MATERIALS AND METHODS

### PIV5 strains.

Genome sequences were determined for strains DEN, RQ, LN, MEL, and MIL (6); W3 (1) (kindly provided by Robert Lamb, Northwestern University, USA); 78524 and H221 (kindly provided by Oswald Jarrett, University of Glasgow, United Kingdom); SER (14) (kindly provided by Hans-Dieter Klenk, Philipps Universität, Marburg, Germany); and CPI^+^ and its variant CPI^−^ (15, 16). Details of these viruses are given in Table 1.

### Preparation and sequencing of PIV5 RNA.

Viruses were grown in Vero cell monolayers at 37°C in Dulbecco's modified Eagle's medium supplemented with 10% fetal bovine serum (17). Cells grown in roller bottles were infected with PIV5 at an MOI of 10 PFU/cell. At 2 days postinfection, nucleocapsids in the form of ribonucleoproteins were purified from infected cells by a series of centrifugation steps, and genomic RNA was isolated, as described previously (17).

Viral RNAs were reverse transcribed, and the DNA was sequenced by standard approaches, using an Illumina GAIIx instrument. Reads with a length of 73 nt were aligned initially to the published strain W3 genome sequence (GenBank accession number AF052755) by using Maq (18), and the assembly was viewed by using Tablet (19). The reads for each strain were then aligned to the appropriate, derived consensus sequence, and the final assemblies were confirmed by inspection. Details of the read data and the GenBank accession numbers of the sequenced genomes are listed in Table 1.

Data on single-nucleotide polymorphisms (SNPs) within each genome sequence were extracted by using Maq, assessed to ensure their representation on both strands of the DNA sequence, and checked by viewing the alignments. The percentage of genomes containing each SNP was calculated by using a script (read_cleaner; D. Gatherer, unpublished data) to count the numbers of reads containing the variant nucleotide flanked on each side by 10 nt (20). In a few instances, adjustments were made to the sequence or positioning of this 21-nt region in order to cater for the location of SNPs close to each other or near the 3′ end of the genome.

The published genome sequences of PIV5 strains W3 (21), cryptovirus, 1168-1, D277, 08-1990, and KNU-11 (22) were obtained from GenBank (Table 1).

### Bioinformatic analyses.

Sixteen of the sequences listed in Table 1 were aligned by using ClustalW implemented in MEGA 5.2 (23); that of the original version of strain W3 [W3(cl)] (see below) was excluded. A maximum likelihood tree was drawn in MEGA by using a general time-reversible, gamma-distributed (GTR+Γ) nucleotide substitution model. Nucleotide substitution was quantified in DNASp (24) by using the Jukes-Cantor substitution model over a sliding window of 500 nt. Codeml, as implemented in the PAML suite (25), was used to identify residues that have potentially evolved under positive selection and to calculate the ratio of nucleotide transitions to transversions (κ). Sequence distance matrices were calculated in MEGA by using the maximum compositional likelihood model with a gamma rate variation distribution.

### Immunological detection of PIV5 SH.

Vero cell monolayers in 25-cm^2^ plates were infected with strain W3, CPI^+^, CPI^−^, or SER at an MOI of 10 PFU/cell. Cell lysates were prepared at 16 h postinfection, and proteins were separated by SDS-PAGE and transferred onto nitrocellulose membranes. Membranes were probed by using a polyclonal peptide antiserum against strain W3 SH (kindly provided by Biao He, University of Georgia, USA), a monoclonal antibody against strain LN P (SV5-Pe) (26), or a monoclonal antibody against cellular β-actin (catalog number A5441; Sigma). Bound antibody was revealed by incubation with horseradish peroxidase-conjugated secondary antibody (catalog number A9169 or A9044; Sigma) and visualized by enhanced chemiluminescence (catalog number 80196; Pierce).

### Analysis of PIV5 diversity during passage *in vitro*.

Previously, we generated PIV5 stocks rich in defective interfering (DI) particles by passaging at a high MOI (17). Briefly, Vero cell monolayers in 75-cm^2^ flasks were infected at an MOI of 5 PFU/cell with a low-passage-number stock of strain W3 (W3vM0). The culture medium was harvested every 2 to 3 days, and half was then used for analysis and the other half was used to infect another flask of cells. Twelve passages of strain W3 (W3vM1 to W3vM12) were performed. To monitor pseudoreversion of PIV5VΔC (originally called rSV5VΔC [27]), the virus was passaged 6 times in Vero cells from a working stock to generate PIV5VΔCp6.

RNA was isolated from purified nucleocapsids from the later strain W3 passages (W3vM8 to W3vM12), and sequence data were derived as described previously (17). Reads with a length of 73 nt were aligned to the W3vM0 sequence (GenBank accession number JQ743318) (Table 1), and the alignments were visualized by using Tablet. Similarly, reads with a length of 76 nt obtained from PIV5VΔCp6 RNA were aligned to the PIV5VΔC genome sequence. We have utilized the sequence data for W3vM0 and W3vM12 to characterize the DI populations in these virus stocks (17). SNPs were identified, and the proportions of variant nucleotides were determined, as described above. SNPs in a small region of the V open reading frame (ORF), in which pseudoreversion occurred, were investigated by identifying reads containing the flanking sequences and counting the numbers containing various combinations of variants in the intervening sequence.

### Nucleotide sequence accession numbers.

The sequences determined in this study were deposited in the GenBank database under accession numbers JQ743318 to JQ743328.

## RESULTS

### Sequence relationships among PIV5 strains.

We generated a total of 11 new PIV5 genome sequences by deep sequencing of genomic RNA isolated from purified nucleocapsids derived from infected cells (Table 1). The consensus sequences of the newly analyzed PIV5 strains were compared among each other and the six PIV5 sequences available from GenBank (Table 1). The newly derived sequence for strain W3 was generated from an uncloned virus stock that we originally obtained from Robert Lamb (Northwestern University, USA). It differs from the sequence deposited previously in GenBank (21), which was derived from cloned cDNA and which we refer to as W3(cl), by four synonymous substitutions (nt 505, 5882, 8164, and 9844) and two nonsynonymous substitutions (nt 4210 in M, resulting in G rather than V at residue 357, and nt 4599 in F, resulting in T rather than A at residue 24). From comparisons with the other strains, it was apparent that the differences at nt 505, 4210, 5882, 8164, and 9844 are unique to the strain W3(cl) sequence. The difference at nt 4599 is unique to the newly derived W3 sequence and is also represented as an SNP, with the minor variant being identical to the W3(cl) sequence. From this point in the study, we used only the new consensus sequence for strain W3. The length of the alignment of the 16 genome sequences was 15,252 nt, of which 1,192 positions (7.82%, including a 6-nt insertion in some strains) were variable. Among the variable positions, 842 positions represent differences in >1 sequence and are phylogenetically informative. Consistent with the level of positional variability over the entire alignment, the average pairwise difference is 2.1%, and no two sequences are >5% divergent (Table 2). The most divergent pair are the human strain LN and the canine strain D277 (Table 2 [taking into account additional decimal places not shown]).

**TABLE 2:** Data from pairwise comparisons of complete PIV5 genome sequences^*a*^

Strain	No. of substitutions/nt site or ω value															
	MEL	MIL	DEN	LN	RQ	W3	Crypto	SER	KNU-11	CPI^+^	CPI^−^	1168-1	78524	H221	08-1990	D277
MEL		**1.25**	**2.40**	0.99	**1.73**	0.13	0.13	0.20	0.22	0.13	0.14	0.12	0.10	0.11	0.13	0.13
MIL	0.001		**1.13**	0.80	**1.25**	0.13	0.13	0.19	0.22	0.13	0.14	0.12	0.09	0.10	0.13	0.13
DEN	0.001	0.001		0.64	**1.40**	0.12	0.12	0.18	0.21	0.13	0.13	0.11	0.09	0.10	0.12	0.12
LN	0.002	0.001	0.001		0.00	0.13	0.13	0.20	0.23	0.14	0.14	0.12	0.09	0.10	0.13	0.13
RQ	0.001	0.001	0.001	0.000		0.13	0.13	0.20	0.23	0.14	0.14	0.12	0.09	0.10	0.13	0.13
W3	0.015	0.015	0.014	0.015	0.015		0.21	0.44	0.43	0.21	0.23	0.22	0.13	0.15	0.15	0.15
Crypto	0.026	0.025	0.025	0.025	0.025	0.017		0.27	0.31	0.16	0.17	0.16	0.12	0.13	0.14	0.14
SER	0.020	0.020	0.019	0.020	0.020	0.011	0.018		0.89	0.25	0.28	0.35	0.17	0.19	0.17	0.16
KNU-11	0.022	0.022	0.022	0.023	0.022	0.014	0.020	0.005		0.28	0.29	0.39	0.20	0.22	0.18	0.18
CPI^+^	0.029	0.029	0.029	0.029	0.029	0.021	0.026	0.017	0.019		**1.62**	0.18	0.12	0.14	0.14	0.14
CPI^−^	0.030	0.030	0.029	0.030	0.030	0.022	0.027	0.017	0.020	0.001		0.19	0.13	0.14	0.14	0.14
1168-1	0.020	0.020	0.019	0.020	0.020	0.011	0.018	0.009	0.012	0.018	0.019		0.11	0.13	0.16	0.15
78524	0.027	0.027	0.026	0.027	0.027	0.020	0.025	0.017	0.020	0.026	0.027	0.013		0.28	0.14	0.13
H221	0.027	0.027	0.026	0.027	0.026	0.019	0.025	0.017	0.020	0.026	0.027	0.013	0.001		0.14	0.14
08-1990	0.044	0.044	0.043	0.044	0.044	0.036	0.043	0.032	0.036	0.041	0.042	0.030	0.028	0.027		0.11
D277	0.045	0.044	0.044	0.045	0.045	0.037	0.043	0.033	0.036	0.042	0.042	0.030	0.028	0.028	0.003	

a The bottom left half shows the number of substitutions per nucleotide site. The top right half shows ω values, with high values in boldface type. Crypto, cryptovirus.

**FIG 1:**
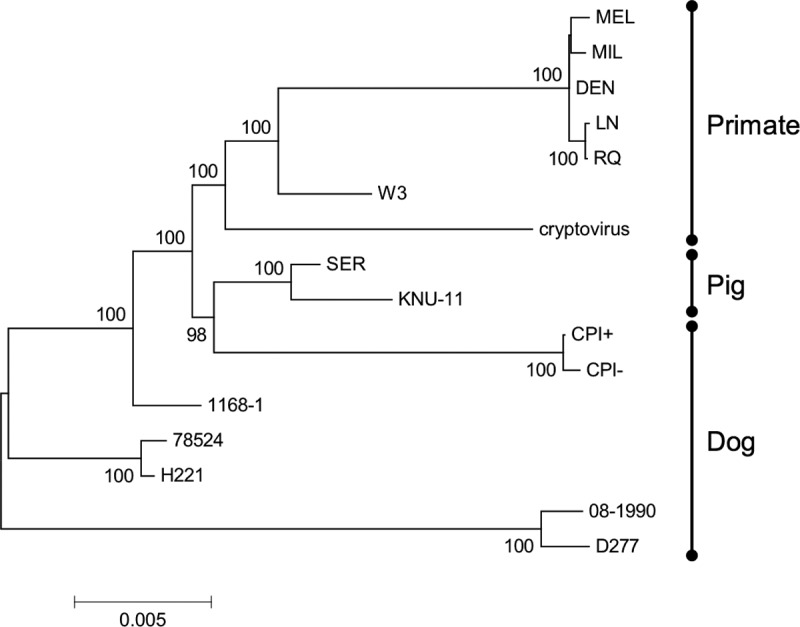
Maximum likelihood phylogenetic tree derived from an alignment of 16 complete PIV5 genome sequences, rooted at the midpoint. Bootstrap confidence values of >70% (from 100 tests) are indicated. The scale indicates the number of substitutions per site.

A phylogenetic tree based on an alignment of the 16 genome sequences is shown in Fig. 1. The primate strains cluster together with 100% confidence, with the human cryptovirus sequence being the outlier in this group. The two porcine strains SER and KNU-11, which were isolated in Germany and South Korea, respectively (Table 1), are closely related to each other, with 100% confidence, and cluster with 98% confidence with the canine viruses CPI^+^ and CPI^−^, which were isolated in Germany (Table 1). Strain CPI^−^ is formally a variant of strain CPI^+^, which was isolated from the brain of a dog with temporary posterior paralysis and caused acute encephalitis when injected intracranially into gnotobiotic dogs (16). The variant was isolated by those authors from one such experimentally infected dog at 12 days postinfection. Unlike strain CPI^+^, variant strain CPI^−^ fails to block interferon (IFN) signaling because of 3 amino acid substitutions in V, leading to a loss of the ability to target STAT1 for proteasome-mediated degradation (28). The greatest genetic diversity is apparent among the canine strains, which were isolated in Germany, South Korea, and the United Kingdom (Fig. 1 and Table 1). However, these strains cluster separately from the primate strains. Registering the caveat that confidence is <70% at the deepest nodes, the longest path is between the cluster of human strains and two of the South Korean canine strains (08-1990 and D277). In interpreting the data on sequence relationships, we cannot rule out the possibility that a degree of convergence may have occurred during propagation of the strains in Vero cells, which are unable to produce IFN. However, even if this were the case, it seems unlikely that it was significant, as host-dependent phylogenetic clustering of isolates was observed.

### Sequence diversity among PIV5 strains.

**FIG 2:**
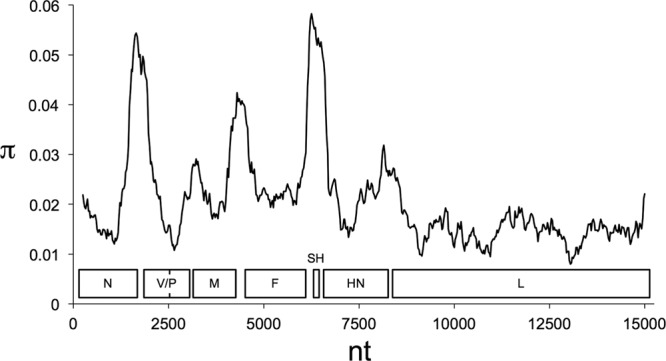
Nucleotide substitutions per site (π) measured in a 500-nt sliding window along an alignment of 16 complete PIV5 genome sequences. Values are plotted to correspond to the center of the window. The arrangement of ORFs is indicated.

In addition to illuminating the relationships among PIV5 strains, the sequences also provide information on the extent of variation along the genome, thus potentially yielding insights into regions in which this factor may relate to function. Diversity was analyzed in terms of nucleotide substitutions per site, expressed as π (29), and was generally highest at gene junctions. In particular, three regions of relatively high variation were identified as peaks (Fig. 2). The first centers on nt 1700 and represents the C-terminal region of N (approximately residues 416 to 509), the 3′ untranslated region (UTR) of the N mRNA, the 5′ UTR of the V/P mRNA, and the N-terminal region of V and P (residues 1 to 76). The second centers on nt 4400 and represents the C terminus of M (residues 366 to 377), the 3′ UTR of the M mRNA, the 5′ UTR of the F mRNA, and the N terminus of F (residues 1 to 24). The third centers on nt 6300 and represents the 3′ UTR of the F mRNA, the SH gene (examined in more detail below), and the 5′ UTR of the HN mRNA.

A region of relatively moderate variation centers on nt 8300 and maps to the C-terminal region of HN (residues 524 to 565) and the 3′ UTR of the HN mRNA (Fig. 2). Since, owing to immune selection, variation might be expected in the viral glycoproteins, the amino acid substitutions in HN were scrutinized. Unique residues are apparent in certain groups of strains, such as most or all of the primate strains (L22, S49, G57, A254, S318, T460, and T536), the primate and porcine strains (D447), and the porcine strains (N120, T288, and S524). However, the level of variation observed overall is modest and not notably greater than that in other PIV5 proteins. Thus, although the substitutions observed might reflect a degree of selection for antibody-mediated evasion, the evidence is not compelling. A similar conclusion was drawn previously for F (7), and our more extensive analysis did not reveal any additional insights, with the exception that the E132K change, which was observed previously only in nonprimate strains, is not present in canine strain 1168-1. Also, strain W3 has seven substitutions absent from HN in other strains (A10, S114, M148, F209, G312, R368, and T491), which may be the result of extensive laboratory adaptation or indicative of a simian origin. A second simian isolate is required to distinguish between these two possibilities.

Three areas of relatively low variation are also evident (Fig. 2). The first centers on nt 2600 and corresponds to the C-terminal regions of P and V (residues 165 to 351 and 165 to 222, respectively). This part of the genome contains the overlapping protein-coding regions of the V/P gene and the part of the P gene encoding residues that interact with L (30). In the part of the genome that encodes L, two regions are particularly well conserved. These regions encompass approximately nt 10000 to 11200, which encode the region (amino acid residues 716 to 931) responsible for RNA-dependent RNA polymerase activity (31), and nt 12800 to 13500 (residues 1463 to 1696), which are of undetermined function.

All of the variation among PIV5 strains occurs as substitutions, except for a 6-nt insertion in the region between the SH- and HN-coding regions in strains LN and RQ, which are closely related to each other (Fig. 1 and Table 2). In most strains, the SH transcriptional termination site is located after nt 6502 to 6515, which (in the antigenome or mRNA sense) has the sequence 5′-UUUUAAAGAAAAAA-3′, followed by an intergenic U residue. Strains LN and RQ have an extended poly(A) site in this region (5′-UUUUAAAGAAAAAGAAAAAA-3′ [the insertion is underlined]). The functional significance of this additional sequence is not known. However, the size of the insertion ensures that the strain LN and RQ genomes, like those of the other strains, adhere to the rule of six, whereby the genome sizes of certain paramyxoviruses occur in multiples of 6 nt (32).

In order to analyze the mode of PIV5 evolution, pairwise comparisons of the ratio of nonsynonymous to synonymous nucleotide substitutions (ω) were made (Table 2). A ω value of 0 indicates that nonsynonymous substitutions have been completely suppressed, a ω value of 1 indicates that neutral accumulation of substitutions has occurred, and ω values of >1 indicate that positive selection may have taken place. Except in a few instances in which sequences are very closely related and ω values are statistically unsound as a result (data in boldface type in Table 2), ω values for PIV5 strains isolated from various hosts are well below 1 (averaging 0.19), indicating that moderate to strong constraint has been the prevailing selective mode. Values of ω for individual ORFs (Table 3) range from indicating strong selective constraint in L to indicating near neutrality in SH. An analysis of positive selection of specific amino acid residues revealed none at a significance level of a *P* value of <0.01, and three candidate residues (residues 447 in HN, 60 in M, and 31 in SH) were detected at a lower significance level (*P* < 0.05). However, we consider that the evidence for positive selection of specific PIV5 proteins, or residues therein, is not compelling.

**TABLE 3:** Values of ω and κ in 16 PIV5 genomes for individual ORFs

ORF	Size (nt)	ω	κ
NP	1,530	0.13	11.99
V	669	0.32	11.90
P	1,179	0.38	9.67
M	1,134	0.15	6.64
F	1,590	0.29	7.68
SH	135	0.87	21.31
HN	1,698	0.24	5.71
L	6,768	0.11	7.26

The ratio of nucleotide transitions to transversions (κ) for individual ORFs is shown in Table 3. The κ value for the combined protein-coding sequences is 8.17, which is somewhat higher than that found thus far for other viruses in the subfamily Paramyxovirinae, such as measles virus, for which the κ value is 5.1 (33, 34). This overall value may have been enhanced by the high value for SH, for which special considerations may apply (as discussed below). The significance of the variation in κ for the other genes is not known.

As well as providing a view of diversity among the consensus PIV5 sequences, the sequence data also yielded information on variation within each of the 11 newly sequenced strains, detectable as SNPs, which were recorded when the minor variant was present in >2% of the sequence population (Table 4). The total number was 111, and the number in each genome ranged from 0 in strain MEL to 19 in strain DEN. With the exception of one SNP, it was possible to infer from the genome sequence alignment which of the two variant nucleotides at each SNP most likely corresponds to the original and which corresponds to the mutation. Most minor variants (93/110; 85%) were inferred as representing mutations, and the proportions of instances in which this is not the case varied from genome to genome (e.g., they were more common in strain MIL than in strain DEN). Among the SNPs located in the ORFs, most (73/103; 71%) are nonsynonymous, perhaps indicating that the cognate variants may have been selected to detectable levels because they conferred a growth advantage. Only a single instance of an SNP shared among strains was discovered (nt 10755 in strains DEN and H221). It is notable that many of the SNPs in strain MIL map in the region at nt 1326 to 1950, which corresponds to the first peak of variation among strains (Fig. 2). There appear to be an underrepresentation of SNPs in the M ORF (observed/expected = 0.38; *P* = 0.05) and an overrepresentation in the HN ORF (observed/expected = 1.62; *P* = 0.02). The other ORFs and the nontranslated regions are represented as expected from a random distribution of SNPs.

**TABLE 4:** SNPs in the PIV5 genome sequences determined in this study

Strain^*a*^	Position (nt)^*b*^	Major variant^*c*^	Minor variant^*c*^	Minor variant frequency (%)	ORF(s) or region affected	Coding difference(s)^*d*^
MIL	71	a	G	8	N 5′ UTR	None
	1462	c	U	30	N	None
	1493	c	U	31	N	H448Y
	1511	U	c	17	N	Y454H
	1601	c	U	26	N	None
	1645	U	c	26	N	None
	1715	U	c	13	V/P 5′ UTR	None
	1746	U	c	12	V/P 5′ UTR	None
	1764	U	c	12	V/P 5′ UTR	None
	5544	C	a	6	F	Q339K
	6884	u	C	33	HN	F101L
	6885^*e*^	U	c	10	HN	F101P
	11799	A	g	5	L	K1129R
	13697	u	C	10	L	Y1762H
DEN	1471	A	g	6	N	None
	1561	U	c	2	N	None
	1650	U	c	43	N	L500P
	1737	U	c	8	N 3′ UTR	None
	3620	A	g	42	M	I160M
	4218	A	c	3	M	N360H
	5840	A	g	42	F	None
	7216	A	g	8	HN	None
	7698	C	u	45	HN	T372I
	9662	G	u	24	L	V417L
	10755	C	u	14	L	T781I
	10837	A	g	8	L	None
	12406	A	g	18	L	None
	12463	A	c	6	L	None
	12491	U	c	3	L	F1360L
	13810	A	g	33	L	None
	14421	C	u	25	L	A2003V
	14723	A	g	38	L	R2104G
	14982	U	c	8	L	L2190S
LN	5259	A	g	5	F	I244V
	6938	u	C	42	HN	None
	14975	U	c	2	L	None
RQ	14291	A	g	8	L	T1958A
	14965	U	c	5	L	None
W3	25	u	C	47	Leader	None
	4599	a	G	25	F	T24A
	5807	C	u	5	F	None
	5879	C	u	15	F	None
	7702	U	g	29	HN	N373K
	14927	A	g	20	L	N2172D
PIV5VΔCp6^*f*^	25	u	C	44	Leader	None
	9356	C	u	34	L	H315Y
	11870	c	U	48	L	None
SER	570	C	u	10	N	T140I
	1227	g	A	48	N	R359K
	4709	G	c	12	F	None
	8201	A	c	19	HN	I540L
	9191	U	c	39	L	Y260H
	9658	U	a	9	L	H415Q
	9995	G	u	9	L	V528L
	10356	A	g	3	L	D648G
	11598	U	c	5	L	I1062T
	14156	C	U	11	L	R1915C
CPI^+^	1520	G	a	6	N	E457K
	1891	U	c	5	V, P	None
	1925	U	c	6	V, P	Y26H
	1998	U	c	6	V, P	L50P
	2395	U	c	14	V, P	None, M183T
	2618	A	g	4	P	I257M
	5364	A	g	15	F	T279A
	6708	A	g	5	HN	Q42R
	6878	A	g	14	HN	K99E
	7224	A	g	12	HN	K214R
	8142	A	g	8	HN	E520G
	9105	C	u	16	L	T231I
	9184	U	c	11	L	None
	10135	U	g	16	L	None
	12492	U	c	16	L	F1360S
	12529	A	c	8	L	None
	13390	C	u	11	L	None
	14775	a	G	15	L	D2121G
CPI^−^	1435	U	c	4	N	None
	6889	A	c	11	HN	Q102H
	7128	U	a	6	HN	L182Q
	7803	A	c	26	HN	N407T
	8141	G	a	6	HN	E520K
	10513	A	u	22	L	None
	11896	A	g	5	L	None
	13318	U	a	11	L	None
	15221	u	G	46	Trailer	None
78524	3382	G	a	4	M	R81K
	5620	G	a	9	F	S364N
	5862	G	a	10	F	D445N
	7145	C	u	43	HN	H188Y
	7146	A	g	24	HN	H188R
	7679	C	u	11	HN	P366S
	10765	A	g	9	L	None
	13712	A	g	7	L	N1767D
	13731	C	u	15	L	T1773I
	14159	G	a	8	L	A1916T
	14189	C	u	2	L	P1926S
	14313	U	g	10	L	F1967C
	14355	C	a	6	L	A1981D
	14360	C	u	14	L	H1983Y
	14503	C	a	4	L	H2030Q
	14506	A	u	14	L	L2031F
	14532	C	u	4	L	A2040V
	14993	U	g	3	L	Y2194D
H221	2319	c	U	4	V, P	S157F
	2765	a	U	5	P	K306N
	4295	C	a	5	M 3′ UTR	None
	7023	A	c	4	HN	N147T
	7058	a	G	4	HN	K159E
	9712	A	g	13	L	None
	10755	C	u	3	L	T781I
	12400	A	g	3	L	None
	13032	C	a	13	L	P1540Q
	13703	U	g	5	L	F1764V
	13874	u	C	4	L	None
	14349	a	G	46	L	D1979G

a No SNPs were detected in strain MEL.

b In the genome sequence of the relevant strain.

c The original nucleotide inferred from the alignment of PIV5 genome sequences is shown in uppercase type, and the mutant nucleotide is shown in lowercase type. In the single instance where an inference could not be made (nt 14156 in strain SER), both nucleotides are shown in uppercase type.

d The amino acid residue in the major population is shown to the left of the position in the relevant protein, and that in the minor population is shown to the right. Note that the original nucleotide may correspond to the major or minor population (see above).

e The minor variant at nt 6885 is linked to that at nt 6884.

f The four clustered SNPs associated with pseudoreversion in the V ORF are not listed and are instead described in the text.

### Functional analysis of the PIV5 SH gene.

**FIG 3:**
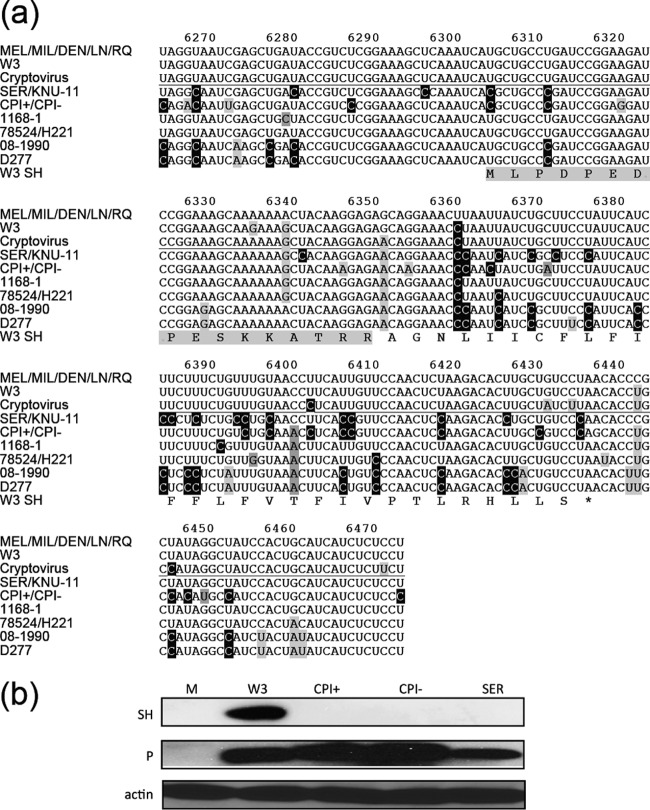
Effects of variation in the PIV5 SH gene on expression of SH. (a) Alignment of genome sequences (in the antigenome or mRNA sense) in the region at nt 6264 to 6473. The line divides primate strains (top) from nonprimate strains (bottom). U-to-C transitions, other transitions, and transversions are shaded black, light gray, and dark gray, respectively, generally for nonprimate strains in relation to primate strains. The amino acid sequence of strain W3 SH is also shown, with the peptide against which the SH antibody was used in panel b shaded light gray. (b) Immunoblot of mock-infected cells (M) and cells infected with PIV5 strains W3, CPI^+^, CPI^−^, and SER, probed with antibodies against PIV5 SH, PIV5 P, and cellular actin.

As described above, one of the regions of relatively high variation maps to the SH gene (Fig. 2). This is due largely to a high level of U-to-C substitutions in the genomes of most nonprimate strains relative to those of primate strains (Fig. 3a). In this region, the primate strains may be placed into three groups, since the human strains MEL, MIL, DEN, LN, and RQ are identical. The nonprimate strains fall into six groups, since the porcine strains SER and KNU-11 are identical, as are the canine strain CPI^+^ and its variant CPI^−^ and the canine strains 78524 and H221. The canine strains 08-1990 and D277 differ at a single nucleotide. Relative to strain MEL/MIL/DEN/LN/RQ, various numbers of the 60 U residues in this region are replaced by C residues in the other strains, as follows: 1 in W3, 3 in cryptovirus, 23 in SER/KNU-11, 20 in CPI^+^/CPI^−^, 2 in 1168-1, 3 in 8524/H221, and 21 in 08-1990/D277.

One of the U-to-C transitions in strains CPI^+^/CPI^−^ and SER/KNU-11 is located in the SH AUG initiation codon, resulting in ACG. Moreover, the UAA termination codon is also mutated, appearing as CAG in strain CPI^+^/CPI^−^ and as CAA in strain SER/KNU-11. The hypothesis that SH is not expressed by these strains, as was adduced in relation to strain KNU-11 (22), was tested by immunoblotting of lysates of Vero cells infected with strain W3, strain CPI^+^, variant strain CPI^−^, or strain SER using a polyclonal peptide antiserum raised against the N-terminal 16 residues of strain W3 SH. The sequence of this region is well conserved among strains (Fig. 3a), with the peptides in strains SER and CPI^+^/CPI^−^ differing from those in strain W3 by 0 and 1 residues, respectively (not including the first residue). The results indicate that strain W3 expressed SH, whereas strain CPI^+^, variant strain CPI^−^, and strain SER did not express an SH-related protein of a similar size (Fig. 3b) or larger (data not shown).

### Sequence diversity of PIV5 passaged in cell culture.

To determine whether the relative stability of the PIV5 genome observed among strains is also a feature of the virus passaged *in vitro*, the strain W3 genome was examined during sequential passaging at a high MOI in Vero cells. We generated this passage series previously, in order to analyze the production of PIV5 DI genomes (mostly of the trailer copyback variety), which are powerful inducers of the interferon response (17). Sequence data were obtained for the input virus (W3vM0; the same data were used to obtain the new sequence of strain W3) (Table 1) and passages 8 to 12 in the series (W3vM8 to W3vM12). The major DI genome in W3vM12 accounted for 94% of all trailer copyback genomes (the major variety of DI genome) and was identified previously as resulting from the joining of nt 15062 (on the 5′-to-3′ strand) to nt 14496 (on the 3′-to-5′ strand) (17). The proportion of non-DI genomes in the population was estimated by aligning the reads separately to the nondefective and defective regions of the genome and deriving average read coverage values. This analysis indicated that W3vM8 to W3vM12 consisted of approximately 89, 86, 89, 94, and 98% DI genomes, respectively. For this exercise, W3vM0 was assumed essentially to lack DI genomes, and indeed, no reads representing the novel junction in the major DI genome were detected.

A total of 11 SNPs reached an abundance of >5% in at least one of the passages monitored (Table 5). Replacement of the U residue at nt 14 by C was linked to replacement of the C residue at nt 25 by U. These SNPs are located in the promoter at the 3′ end of the genome, and strikingly, the emerging variants dominated the viral population in W3vM12. Some of the other SNPs present in W3vM0 increased in abundance (at nt 2482, 10442, 11967, and 13261), and some decreased (at nt 4599, 5807, 5879, 7702, and 14927; the last was present in the major DI genome, and it was not possible to ascertain whether it was also present in the non-DI genome population). These results show that, even over 12 passages *in vitro* and under conditions where nondefective genomes were subjected to competition from an overwhelming proportion of DI genomes, the nondefective sequences exhibited little diversity.

**TABLE 5:** SNPs during passage of PIV5 strain W3

Position (nt)	Major variant^*a*^	Minor variant^*a*^	ORF(s) or region affected	Coding difference(s)^*b*^	Kinetics^*c*^	Minor variant frequency (%)^*d*^					
						W3vM0	W3vM8	W3vM9	W3vM10	W3vM11	W3vM12
14	U	c	Leader	None	I	0.00	93.88	97.43	97.67	99.51	98.28
25	u	C	Leader	None	D	47.03	0.00	0.44	0.33	0.21	0.43
2482	U	c	V, P	None, V212A	I	0.11	4.45	7.89	13.45	16.37	19.35
4599	a	G	F	T24A	D	24.77	0.18	0.44	0.15	0.31	0.29
5807	C	u	F	None	D	5.28	0.03	0.00	0.00	0.04	0.00
5879	C	u	F	None	D	14.91	0.03	0.00	0.07	0.04	0.09
7702	U	g	HN	N373K	D	29.35	2.78	2.21	1.50	1.09	0.74
10442	A	c	L	I677L	I	0.03	4.91	5.85	7.76	8.75	9.84
11967	C	a	L	A1185D	I	0.03	4.64	6.83	7.99	9.36	8.51
13261	A	c	L	L1616F	I	0.02	3.67	7.41	12.28	13.91	13.46
14927	A	g	L	N2172D	D	19.64	12.87	10.53	12.46	7.96	5.31

a The original nucleotide inferred from the PIV5 genome alignment is shown in uppercase type, and the mutant nucleotide is shown in lowercase type.

b The amino acid residue in the major population is shown to the left of the position in the relevant protein, and that in the minor population is shown to the right. Note that the original nucleotide may correspond to the major or minor population.

c I, increasing in proportion during passage; D, decreasing in proportion during passage.

d The numbers of reads obtained (with the proportions that aligned to the strain W3 genome sequence in parentheses) were as follows: W3vM0, 21,156,178 (78%); W3vM8, 3,233,587 (59%); W3vM9, 3,210,385 (30%); W3vM10, 3,473,827 (42%); W3vM11, 3,636,000 (51%); W3vM12, 3,789,267 (37%).

**FIG 4:**
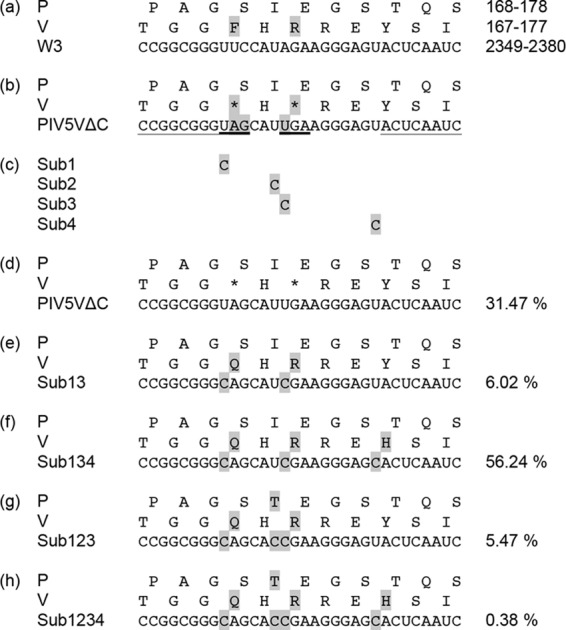
Variation in a region of the PIV5 V/P gene after passaging of PIV5VΔC 6 times in Vero cells to generate PIV5VΔCp6. The number of reads that aligned to the complete PIV5VΔC genome sequence was 22,456,971 (71% of the total). (a) The RNA sequence in W3 and its translation as part of P and V, with coordinates in the genome or amino acid sequences on the right. (b) The RNA sequence in PIV5VΔC and its translation, with substitutions shaded gray and introduced termination codons in the V gene heavily underlined. The light underlining indicates the flanking sequences that were utilized to isolate reads for analysis of SNPs. (c) The four substitutions (sub1 to sub4) detected in this region of PIV5VΔCp6. (d to h) The five major sequence constellations of sub1 to sub4 (parental PIV5VΔC, sub13, sub134, sub123, and sub1234), with percentages of reads on the right. Nucleotide and amino acid substitutions, in comparison with PIV5VΔC, are shaded gray.

In a further investigation of the stability of PIV5 in cell culture, we utilized PIV5VΔC, which is a mutant derived from a molecular clone of strain W3. In this mutant, two translational termination codons have been introduced into the V ORF via substitutions of 3 nt that do not affect the amino acid sequence of P but result in expression of a truncated version of V lacking the C-terminal region (27) (Fig. 4a and b). Passaging of this virus in various cell lines, including Vero, has been shown to result in rapid mutation to a derivative that expresses full-length V, via substitutions in both of the introduced termination codons (27). To monitor this process of pseudoreversion in greater detail, the virus was passaged 6 times in Vero cells to generate a stock named PIV5VΔCp6. Seven SNPs were detected in PIV5VΔCp6. Three were located outside the V ORF (Table 4), and four were located in the V ORF (nt 2357, 2362, 2363, and 2372). The latter SNPs were U-to-C substitutions (sub1, sub2, sub3, and sub4) and are listed in Fig. 4c. Two (sub1 and sub3) are the same as those reported previously (27). The original sequence plus all arrangements of the four substitutions may result, in principle, in 16 different sequence constellations. However, only five (PIV5VΔC, sub13, sub134, sub123, and sub1234) were detected in >0.2% of PIV5VΔCp6 genomes, together summing to 99.58% (Fig. 4d to h). The two changes (sub1 and sub3) in the termination codons resulted in full-length V containing an amino acid substitution (F170Q), and a third change (sub4) added a second amino acid substitution in V (Y175H), to generate the major variant (sub134) (Fig. 4f). In addition, a substitution in P was detected at lower levels (sub2, as represented in sub123 and sub1234) (Fig. 4g and h). This experiment again demonstrated the relative stability of the PIV5 genome during passage at a high MOI and also showed that adaptation can nonetheless occur rapidly when a selection pressure is exerted. The two substitutions (sub2 and sub4) not associated with pseudoreversion of the termination codons were not detected in a previous experiment (27), and in our experiment, it is possible that they were not selected in their own right but were subsidiary to sub1 and sub3.

## DISCUSSION

We generated 11 genome sequences for 10 PIV5 strains and a variant of one of these strains (Table 1). The incorporation of published genome sequences for other strains facilitated the phylogenetic analysis of a total of 15 strains and the variant. In the tree (Fig. 1), the primate strains cluster together, as do the porcine strains, but there is much greater diversity among the canine strains. Nonetheless, there is remarkable sequence conservation overall, even though the strains originated from a variety of hosts and were isolated from a wide geographical area during a period of several decades (Tables 2 to 4). Surprisingly, the level of variation in HN and F, which are targets for antibody-mediated virus neutralization, was not significantly greater than that observed in most other viral proteins. Amino acid residues 342, 437, and 457 in HN have been linked to promoting resistance to neutralizing antibodies (35). However, with the exception of a Q342K substitution in two of the South Korean canine isolates, variation in these, or intervening, residues was not observed. Overall, these results suggest that antibody-mediated selection has not played a major role in the evolution of the PIV5 strains analyzed, perhaps because the virus is not particularly immunogenic or because cell-mediated immunity is more important in controlling PIV5 infections.

The RNA polymerases of paramyxoviruses, like those of most other RNA viruses, lack proofreading mechanisms and therefore have high error rates, resulting in the continual production of large numbers of mutants during replication. Consequently, when a selection pressure is sufficiently strong, for example, in the case of PIV5VΔC, which lacks a functional V ORF, mutations are generated with facility. In this context, it is striking that the PIV5 genome, as represented by the range of strains studied, exhibits so little diversity. Indeed, even the level of synonymous mutations among strains is impressively low and similar to what has been reported for another rubulavirus, mumps virus (36, 37), and a morbillivirus, measles virus (38). The low level of variation in PIV5 is also similar to that observed in human PIV3 isolates. However, the level of variation observed between human and bovine PIV3 isolates is about six times greater than that observed between PIV5 strains isolated from different species.

The reasons for the low levels of diversity in paramyxoviruses are not understood. Constraints potentially operate at several levels, affecting recognition by innate immune responses or the level and rate of protein translation, including biases against specific dinucleotides (39), biases in codon usage (recently shown to be important in HIV-1 replication) (40), and codon pair context (41, 42). Codon usage appears to be able to exert an unexpectedly large effect on translational efficiency in some microbial organisms (43) and also appears to be virus specific, rather than host specific, in the subfamily Paramyxovirinae (B. K. Rima, unpublished data). Since paramyxovirus genomes are always encapsidated, secondary structures in the genomes or antigenomes are unlikely to provide a significant constraint on synonymous mutations. However, mRNA secondary structure can influence the rate of protein translation and thus affect indirectly the proper folding of nascent proteins (44). Finally, it must be registered that any RNA virus enters the RNA world of the host cell and thus must avoid the formation of double-stranded RNA (dsRNA) structures with complementary cellular RNAs, including microRNAs (miRNAs).

The rule of six (32) appears to be an important factor in the fitness of members of the subfamily Paramyxovirinae. Its relevance is demonstrated in our data by strains RQ and LN, which have an insertion of 6 nt at the 3′ end of the SH gene. Since the SH gene of PIV5 is dispensable for growth in cell culture (21), the effect of this insertion cannot be evaluated easily. Toleration of a certain level of diversity at the 3′ end of rubulavirus mRNAs may be seen in a variation in the mumps virus F gene, which led to the insertion of oligo(G) tracts immediately prior to the poly(A) tail without affecting viral growth *in vivo* (37).

The rule of six has been interpreted as indicating that all nucleotides in a genome must be attached to N molecules in groups of six (32). This, in turn, has established the concept of “phase,” that is, the position of a nucleotide within a specific group of six. The phases (phases 1 to 6) of the 5′ nucleotides of each of the mRNAs in all the PIV5 genomes show strong overall conservation, similar to other paramyxoviruses (45). If phase is an important feature of rubulaviruses, it might operate as a constraint on the occurrence of synonymous mutations. However, most of the variation in PIV5 strains is clustered in three regions between (i) the ORFs of the N and V/P proteins, (ii) the M and F ORFs, and (iii) the F and HN ORFs, including the entire SH gene (Fig. 2). The implication that constraint has operated primarily on protein-coding sequences is consistent with the observation that following the loss of protein function, many synonymous and (potentially) nonsynonymous mutations have accumulated, as observed for the SH gene in strains CPI^+^/CPI^−^ and SER.

Analysis of variation in the PIV5 SH gene indicated a preponderance of U-to-C substitutions in many of the nonprimate strains. As a mechanism, this suggests biased hypermutation due to an adenosine deaminase, RNA-specific 1 (ADAR1)-like activity that deaminates A residues in dsRNA to I residues, which then pair preferentially with C residues in subsequent rounds of replication (46). At some point, mutations in strains CPI^+^/CPI^−^ and SER/KNU-11 have led to a change in the AUG initiation codon for SH to ACG (Fig. 3). Although ACG can act as an initiation codon in certain circumstances, for example, in expression of the Y proteins in Sendai virus (47), in PIV5 strains CPI^+^/CPI^−^ and SER, this change, perhaps in combination with others, has led to a lack of expression of SH. The function of SH is unclear, but recent studies have reported that it inhibits tumor necrosis factor alpha (TNF-α)-induced apoptosis (48–50). In certain cell types, such as bovine kidney (MDBK) cells, PIV5 causes little cytopathic effect, but when MDBK cells were infected with recombinant PIV5 lacking the SH gene, an increased cytopathic effect was observed (48). Our results indicate that the SH gene was lost some time ago, as strains CPI^+^/CPI^−^ and SER were isolated in the 1970s and 1990s, respectively (Table 1), and this absence has been maintained in strain KNU-11, which was isolated in 2011 (22). In regard to strain CPI^+^/CPI^−^, it seems very likely, given the genetic stability of PIV5, that the loss of the ability of variant strain CPI^−^ to block IFN signaling was selected *in vivo*, possibly because IFN-sensitive viruses may be better able to establish prolonged or persistent infections (28).

Our study also shows that PIV5 also exhibits little diversity when passaged in cell culture. Thus, serial passaging of strain W3 at a high MOI, under conditions of competition with large proportions of DI genomes, generated only a modest number of mutations (Table 5). Although it would not be expected that competition with DIs would necessarily lead to the selection of nonsynonymous mutations, it was striking that, despite the fact that the PIV5 genome is not codon optimized, the only synonymous mutations selected were in the region of the leader promoter and that no other synonymous mutations were coselected with these promoter mutations. Similar observations were made for the mutant lacking V function (PIV5VΔC), in which mutations were confined largely to pseudoreversion events in the V ORF (Fig. 4). As has been reported previously (27), it is clear that there is strong selection to remove the termination codons and allow expression of intact V, in which the C-terminal zinc finger motif is the most conserved element in members of the subfamily Paramyxovirinae. Pseudoreversion occurred even though the virus was passaged in Vero cells, which are not able to induce IFN. Thus, the pressure to regain V function cannot be related to blocking of the IFN response and is presumably due to a restoration of some other important function of V, for example, in controlling viral transcription or replication. A number of mutations could potentially have arisen in PIV5VΔC that would have led to the loss of the termination codons, but the only pseudorevertants detected in our study and in a previously reported experiment (27) involved replacement of U by C residues (sub1 and sub3). In our experiment, two other U-to-C transitions also occurred closely adjacent to the termination codons (sub2 and sub4). These findings again suggest the operation of biased hypermutation, although (as discussed above for SH) an intrinsic bias of the RNA polymerase cannot be ruled out.

In conclusion, the sequences of PIV5 strains indicate that the genome exhibits little diversity *in vivo* and *in vitro*. However, the identity of the main animal reservoir of PIV5 remains unclear. SH appears to be dispensable for growth in dogs and pigs (although further experimentation will be needed to the determine the consequences, if any, of the loss of SH on PIV5 pathogenesis in dogs and pigs), and the absence of SH from some strains isolated from these animals may imply that they are not the natural hosts. Moreover, the fact that PIV5 is not known to cause detectable disease in humans, whereas infections in dogs may be severe, could be interpreted as indicating that humans are the reservoir host from which PIV5 has transferred into dogs and pigs. If so, this might explain why monkeys brought into captivity apparently become infected by PIV5 through contact with humans. To resolve the question of the origins of PIV5, many more strains from various sources need to be analyzed, particularly from geographical locations where dogs are not immunized against PIV5.

Finally, the findings based on the sequence information presented here raise questions that will form the basis of a number of future mechanistic studies, including (i) investigating the role of ADAR and the mechanisms that underlie biased hypermutation in viruses with encapsidated genomes; (ii) determining why there are relatively few synonymous mutations in viruses isolated over decades and from different species, given that the PIV5 genome is not codon optimized; (iii) elucidating what the selection pressures are on PIV5VΔC to make it revert so quickly in the absence of a host cell IFN response; and (iv) determining why antibody-mediated immune selection appears not to be a major driving force in PIV5 evolution.
